# Mutation screening of *GRIN2B* in schizophrenia and autism spectrum disorder in a Japanese population

**DOI:** 10.1038/srep33311

**Published:** 2016-09-12

**Authors:** Yuto Takasaki, Takayoshi Koide, Chenyao Wang, Hiroki Kimura, Jingrui Xing, Itaru Kushima, Kanako Ishizuka, Daisuke Mori, Mariko Sekiguchi, Masashi Ikeda, Miki Aizawa, Naoko Tsurumaru, Yoshimi Iwayama, Akira Yoshimi, Yuko Arioka, Mami Yoshida, Hiromi Noma, Tomoko Oya-Ito, Yukako Nakamura, Shohko Kunimoto, Branko Aleksic, Yota Uno, Takashi Okada, Hiroshi Ujike, Jun Egawa, Hitoshi Kuwabara, Toshiyuki Someya, Takeo Yoshikawa, Nakao Iwata, Norio Ozaki

**Affiliations:** 1 Department of Psychiatry, Nagoya University Graduate School of Medicine, Nagoya, Aichi, Japan; 2 Institute for Advanced Research, Nagoya University, Nagoya, Aichi, Japan; 3 Brain and Mind Research Center, Nagoya University, Nagoya, Aichi, Japan; 4 Department of Pharmacology, Nagoya University Graduate School of Medicine, Nagoya, Aichi, Japan; 5 Department of Psychiatry, Fujita Health University School of Medicine, Toyoake, Aichi, Japan; 6 Laboratory for Molecular Psychiatry, RIKEN Brain Science Institute, Wako, Saitama, Japan; 7 Center for Advanced Medicine and Clinical Research, Nagoya University Hospital, Nagoya, Aichi, Japan; 8 Department of Psychiatry, Nagoya University Graduate School of Medicine, Nagoya, Japan; 9 Department of Psychiatry, Ujike Nishiguchi Clinic (HU), Okayama, Japan; 10 Department of Psychiatry, Niigata University Graduate School of Medical and Dental Sciences, Niigata, Japan; 11 Department of Pediatric Psychiatry, Center for Transdisciplinary Research, Niigata University, Niigata, Japan; 12 Department of Child Neuropsychiatry, Graduate School of Medicine, The University of Tokyo, Tokyo, Japan

## Abstract

*N*-methyl-d-aspartate receptors (NMDARs) play a critical role in excitatory synaptic transmission and plasticity in the central nervous systems. Recent genetics studies in schizophrenia (SCZ) show that SCZ is susceptible to NMDARs and the NMDAR signaling complex. In autism spectrum disorder (ASD), several studies report dysregulation of NMDARs as a risk factor for ASD. To further examine the association between NMDARs and SCZ/ASD development, we conducted a mutation screening study of *GRIN2B* which encodes NR2B subunit of NMDARs, to identify rare mutations that potentially cause diseases, in SCZ and ASD patients (n = 574 and 152, respectively). This was followed by an association study in a large sample set of SCZ, ASD, and normal healthy controls (n = 4145, 381, and 4432, respectively). We identified five rare missense mutations through the mutation screening of *GRIN2B*. Although no statistically significant association between any single mutation and SCZ or ASD was found, one of its variant, K1292R, is found only in the patient group. To further examine the association between mutations in *GRIN2B* and SCZ/ASD development, a larger sample size and functional experiments are needed.

Schizophrenia (SCZ) is a severe chronic psychiatric disease that is characterized by psychotic symptoms such as hallucinations and delusions. The lifetime risk of SCZ is estimated to be 1%, with subsequent mortality of SCZ patients is 2.5 times higher than in the general population^1^. Autism spectrum disorder (ASD) is characterized by impaired social interactions and communications and by restricted, repetitive behavior. The prevalence of prototypical ASD is around 25 per 10000, and that of broad ASD is 116 per 10000^2^. The heritability of SCZ and ASD is estimated as 60–90% from population-based and twin studies^3^,^4^. Both common and rare genetic variants are associated with the etiology of both disorders^5^,^6^,^7^,^8^. Neurodevelopmental mechanisms and related molecules are strongly involved in both SCZ and ASD^9^.

*N*-methyl-d-aspartate receptors (NMDARs) are ligand-gated ionotropic receptors that play important roles in synaptic plasticity within the central nervous system (CNS). NMDARs consist of two NR1 subunits in addition to either two NR2 (A, B, C, or D) and/or NR3 (A or B) subunits. Two of the four subunits must be NR1, and inclusion of the other two subunits results in multiple combinations^10^. Thus, combinations of subunits result in diversity of physiological and pharmacological functions. Long-term potentiation in the hippocampus depends on NMDAR activity^11^. NMDAR function is involved in CNS activity including cognitive function, behavior, and memory^12^,^13^. These functions are often impaired in psychiatric diseases such as SCZ and ASD. Therefore, NMDARs may contribute to the development of neurodevelopmental disorders.

A number of studies have shown that NMDAR antagonists (e.g., phencyclidine, ketamine) cause psychotic symptoms that are diagnostically difficult to differentiate from SCZ^14^. Moreover, NMDAR encephalitis, which was identified in 2007, has similar symptoms as SCZ. Autoantibodies against the NR1 subunit are produced in these patients, who develop diverse symptoms such as psychosis, memory deficits, seizures, and language disintegration^15^. Several studies suggest that NMDAR abnormalities exist in the CNS of SCZ patients^16^,^17^.

Many studies have reported that dysregulation of NMDARs is implicated in the etiology of SCZ. Mice with reduced expression of NR1 subunits display abnormal SCZ-like behavior that is ameliorated by treatment with antipsychotic drugs^18^. Interactions between the neuregulin 1 receptor (erbB4) and post-synaptic density (PSD)-95 are increased in these mice, and this interaction facilitates activation of erbB4, which suppresses NMDARs^19^. Moreover, d-serine, which acts as an agonist at NMDARs, has therapeutic effects when administered with certain antipsychotic drugs^20^. These findings consistently support the hypofunction hypothesis of NMDARs in SCZ.

Recent studies also suggest that NMDA is implicated in ASD^21^. In an animal model, both excessive and reduced NMDA dysfunctions result in ASD-like phenotypes. Shank2 is a scaffolding protein in the PSD and interconnects receptors at the PSD including NMDARs. Shank2−/− mice have reduced NMDAR activity and also exhibit ASD-like behaviors that are corrected by normalizing NMDAR functions^22^. IRSp53, which is also known as BAIAP2, acts as a scaffolding protein and regulates dendrite spines of excitatory synapses. In contrast to Shank2−/− mice, IRSp53−/− mice have enhanced NMDAR activity in the hippocampus, but also show impaired social interaction. These ASD-like behaviors are rescued by memantine. Thus, NMDAR dysregulation in the CNS may contribute to development of ASD^23^.

A recent genetic study that utilized next generation sequencing technology suggests that NMDARs are strongly implicated in the etiology of both SCZ and ASD. For instance, a large-scale genome-wide association study identified *GRIN2A*, which code NR2A, as an important candidate gene in SCZ susceptibility^24^. Other exome studies reported that *de novo* nonsense mutations and causative variants are found in *GRIN2B*, which code NR2B, in ASD^25^,^26^. In this context, we conducted a study to discover rare single nucleotide variants in *GRIN2B*, since several disease causative variants itself are identified in the same gene. Therefore, we sequenced the exonic regions of *GRIN2B* in SCZ and ASD to detect rare non-synonymous variants, and performed association studies targeting discovered variants in in the current study using large sample set of SCZ, ASD, and healthy controls.

## Materials and Methods

### Subjects

Two independent sample sets were used in this study. The first sample set, comprising 574 SCZ samples and 152 ASD samples, was used for rare missense or nonsense mutation screening. The second sample set, comprising 4145 SCZ patients, 381 ASD patients, and 4432 healthy controls (CON), was used for a genetic association study. Profiles (age and sex) of participants are shown in Table 1.

All participants were recruited in Nagoya University Hospital and its associated institutes and hospitals. All cases were diagnosed according to Diagnostic and Statistical Manual of Mental Disorders, Fifth Edition. In this study, we enrolled patients with either SCZ or ASD. CON participants were evaluated with an unstructured interview to exclude individuals with a history of mental disorders. All participants were ethnically Japanese. Written informed consent was obtained from all subjects. All patients consented to participate in the study. When necessary, a patient’s ability to provide consent was confirmed by his or her guardian. Patients with communication difficulties, or those who did not consent were subsequently excluded. Ethical approval was granted by The Ethics Committee of the Nagoya University Graduate School of Medicine. All experiments were performed in accordance with the Committee’s guidelines and regulations.

### Gene Screening and Variation Analyses

For each sample, we sequenced the coding region of *GRIN2B* (Chr12: 13,537,337–13,980,363 reverse strand, GRCh38 assembly, Ensembl transcriptID ENST00000609686). Genomic DNA was extracted from each subject’s peripheral blood or saliva with standard methods. Sequencing was performed with the standard capillary sequencing method. Primers for target amplification and the dye-termination reaction were designed using FastPCR (PrimerDigital Ltd. Helsinki, Finland) and PerlPrimer respectively^27^. Primer sequences are listed in Supplementary Table 1. Amplification primer was tested using UCSC *in silico* PCR (http://genome.ucsc.edu/cgi-bin/hgPcr) to verify that the intended targets were correct. We used the TAKARA LA Taq polymerase (Takara Bio Inc., Shiga, Japan) for PCR. After PCR amplification, amplicons were processed with Illustra Exonuclease I and Alkaline Phosphatase (GE Healthcare and Life Science, Little Chalfont, United Kingdom) to digest excess primers. The cycle sequencing reaction was then performed with the Big Dye Terminator v3.1 Cycle sequencing kit (Applied Biosystems, Foster City, CA, USA). The reaction products were purified with magnetic beads (CleanSEQ Kit, Beckman Coulter, Krefeld, Germany). Purified products were sequenced on a 3130xL Genetic Analyzer (Applied Biosystems).

Variant Reporter Software (Applied Biosystems) was used for mutation detection analysis. After all variants were screened, we narrowed it down the variants to perform follow-up analyses. Selection inclusion criteria was as follows. (1) Non-synonymous variants including nonsense single nucleotide variants (SNVs), missense SNVs, frame shifts, and small INDELs as candidate pathological variants. (2) We included only rare mutations (minor allele frequency <1%). In each case, the detected variations were validated in a discrete experiment at least once.

### Bioinformatics Analyses

After variant selection, we analyzed variants using the following tools: (1) Human Protein Reference Database (http://www.hprd.org/index_html) and the Pfam database (http://pfam.xfam.org/)^28^ for confirming protein structure and localizations of variants. (2) Polyphen2^29^, Sorting Tolerant from Intolerant (SIFT)^30^, and PMUT^31^ were used to evaluate functional changes arising as a consequence of alteration in amino acid sequence.

### Genetic Association Analyses

For each adopted variant, case-control genotyping studies were performed. We used the Custom Taqman^®^ SNP Genotyping assay (Applied Biosystems) for each variant. DNA samples were prepared on 384-microtiter plates with a positive control. PCR was performed with Taqman^®^ Universal Master Mix 2 with UNG (Applied Biosystems). Allelic discrimination analyses were performed on an ABI PRISM HT7900 sequence detection system (Applied Biosystems). To determine if each detected sample did carry the variant of interest, positive samples were substantiated by at least one further experiment. Failed samples were also reassessed using a second experiment. We omitted samples in which we failed to detect the signals in two experiments.

For the association analysis, statistical tests were performed using JMP Pro (SAS Institute, Cary, NC, USA). Differences in allele and genotype frequencies of the mutations were compared between SCZ patients/controls, ASD patients/controls, and SCZ + ASD patients/controls respectively and significant was calculated using Fisher’s exact test (two-tailed). The threshold of significance set at p < 0.05.

## Results

### Mutation Screening

We identified five rare missense mutations and 14 synonymous mutations within *GRIN2B* coding exons (Table 2 and Supplementary Table 2).

**Figure 1:**
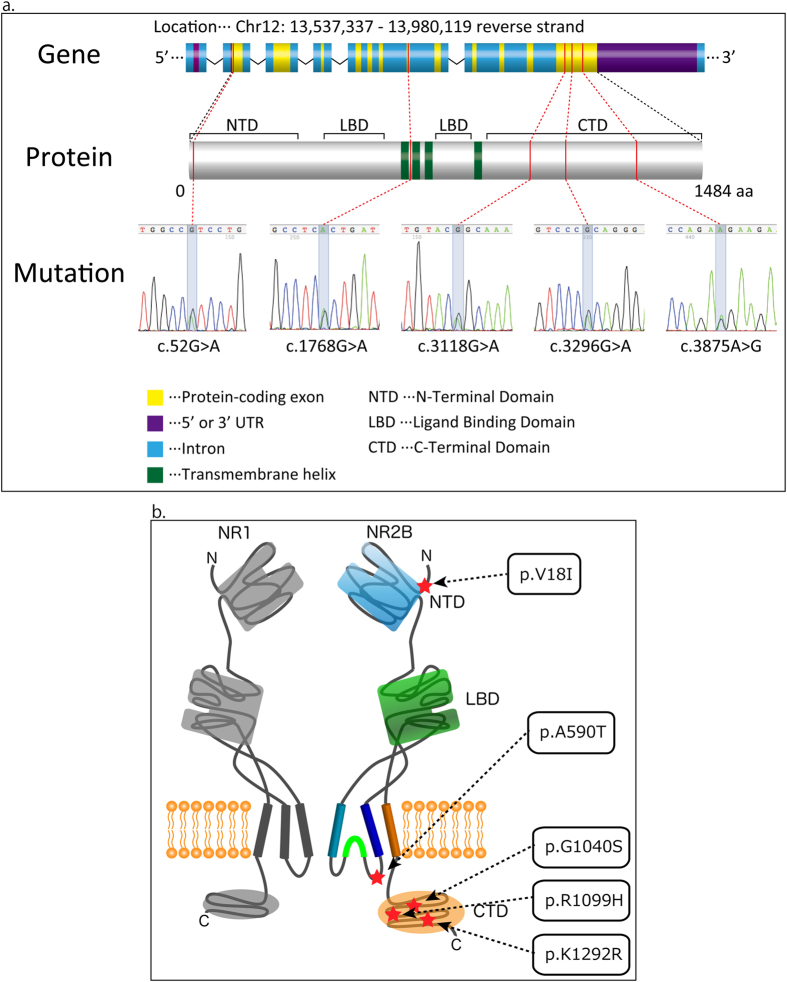
Location of missense variants. (**a**) Variants with respect to the secondary structure of *GRIN2B*. (**b**) This image was created by referring to the crystal structure in the Protein Data Bank (http://www.rcsb.org/pdb/home/home.do. PDB ID: 4TLL)1). The location of each discovered variant (red star) is shown on the image. Columns colored deep blue, green, bright blue, and orange correspond to transmembrane helix1, p-loop, transmembrane helix2, and transmembrane helix3, respectively. Abbreviations: NTD, N-terminal domain; LBD, Ligand-binding domain; CTD, C-terminal domain.

The first mutation was found in the N-terminal domain, the second was found in the ligand-gated ion channel domain, and the other three mutations were within the C-terminal (intracellular) domain (Fig. 1).

We searched three genetic databases (dbSNP build143 (http://www.ncbi.nlm.nih.gov/SNP/index.html), 1000 Genome Project (http://www.1000genomes.org/), and Exome Variant Server (http://evs.gs.washington.edu/EVS/)) to determine if these missense mutations were novel variants. Three missense mutations (V18I, A590T, and G1040S) had already been registered on dbSNP. Additionally, genotype data for A590T were found on the Exome Variant Server. The aforementioned databases, as well as ExAC Browser (http://exac.broadinstitute.org), did not report R1099H and K1292R respectively.

### Bioinformatics Analyses

The five missense variants were analyzed with Polyphen2, SIFT, and PMUT. The overall results are shown in Table 2 along with the screening result. According to the results of these analyses, R1099H was considered to have the most damaging effect due to the substitution in the protein; V18I and K1292R were thought to have a modest effect.

### Results of Genetic Association Analyses

The result of the genotyping experiment is shown in Table 3. All variants except K1292R were detected in the CON group. K1292R was only detected in the SCZ group, and three additional SCZ samples were found to carry K1292R. Using Fisher’s exact test, none of the variants had a significant association with SCZ or ASD.

## Discussion

In summary, we first sequenced the coding region of *GRIN2B* in 574 SCZ and 152 ASD samples using the Sanger sequencing method. Subsequently, we conducted association analysis with a secondary sample set. We detected five rare missense variants through mutation screening. We also sequenced parents’ sample of ASD in screening sample sets, however, we did not detect any *de novo* mutation. Moreover, we could not find significant associations between SCZ/ASD and variants investigated in this study. Although K1292R (Supplementary Result 1) was observed only in SCZ samples, evidence of statistical association, if any, must be obtained in larger population studies.

The amino acid sequence includes an important residue for binding of NR2B to Ca^2+^/calmodulin-dependent protein kinase II (CaMKII)^32^,^33^ (Supplementary Fig. 1). This interaction is key for autoregulation of CaMKII function and synaptic plasticity^31^. Stefan *et al*. previously examined the alteration of the association between NR2B and CaMKII by replacing NR2B residues. They reported that substitution of K (Lysine) 1292 with Q (Glutamine) reduces the affinity of NR2B for CaMKII. In our genetic study, we detected R instead of Q, but we could not examine the effect caused by R substitution. This NR2B-CaMKII protein interaction induces hippocampal long-term potentiation and spatial learning^34^. Of note, in the case of singleton SNVs (variants detected only in one individual or one family), performing association analysis with an extremely large sample size will be needed to achieve statistical significance but will be very difficult. However, the sole fact that the SNV was detected in only case samples is suggestive of its genetic relevance.

### Limitations

There were several limitations in this study that may have influenced our results. Firstly, we used 574 SCZ and 152 ASD cases for mutation detection. Whilst we detected five rare missense mutations that may confer a genetic risk to SCZ/ASD development, due to the size of our screening samples, we may have limited our potential to identify other disease-causing variants. Specifically, the number of variants detected within *GRIN2B* in healthy subjects^10^, indicated that *GRIN2B* is among top 1% of the human genes that are least likely to carry functional genetic variation relative to the genome wide expectation given the amount of apparently neutral variation the gene has. Therefore, future studies may benefit from using much larger sample sizes to increase the likelihood of detecting a larger number of functionally relevant rare single nucleotide variants within *GRIN2B*.

Secondly, we applied stringent filtering criteria to control for multiple testing, because larger number of statistical tests (i.e. larger number candidate SNVs) equals lower (i.e. stringent) p-values which in turn translates into larger sample sizes required to obtain statistical evidence. Therefore, we chose to limit the number of candidate SNVs in order to have realistic chance for detecting statistical association using sample which we had available for this study. It is of note, however, that we have detected K1292R only in cases. Although, for this variant we could not obtain statistically significant results per se (i.e. P-value < 0.05), it is still relevant from biological point of view that this variant was not detected in controls. Considering that we had detected only few number of candidate variants in *GRIN2B* may be indication of its functional significance as the strong negative selection pressure upon amino acid sequence of *GRIN2B* may be related to relative scarcity of variants observed in general population.

Finally, after using all of our genotyping samples, we conducted a power calculation of the statistical power for K1292R^35^. The parameter settings were: High-risk allele frequency, 0.0004; Prevalence, 0.01; Genotype relative risk Aa, 3; Genotype relative risk AA, 9; D-prime, 1; Marker allele frequency, 0.0004; Number of cases: 4145; Control-case ratio, 1.0693; User-defined type I error rate, 0.05; User-defined power, 0.80. According to our power calculation based on the minor allele frequency of genotyping, approximately 9109 SCZ samples are needed to obtain 80% power. In other words, based on our study design, we can achieve 80% power if GRR is larger than 5 under multiplicative model, and we can achieve nearly 99% power if GRR is larger than 7 under same genetic model.

## Conclusion

In conclusion, we found five rare missense mutations with our mutation screening. We also conducted genotyping for each detected variant. Whilst we did not find any significant association between any variant with SCZ or ASD, we found that K1292R was only present within the SCZ samples. Whilst K1292R was considered to confer a vulnerable effect for carriers, further genotyping, using a larger sample size, is needed to better understand its association with SCZ development. Moreover, to comprehensively assess impact of rare variants affecting NMDA signaling in case of psychiatric disorders, genes beyond *GRIN2B* should be evaluated in the future studies.

## Additional Information

**How to cite this article**: Takasaki, Y. *et al*. Mutation screening of *GRIN2B* in schizophrenia and autism spectrum disorder in a Japanese population. *Sci. Rep.* **6**, 33311; doi: 10.1038/srep33311 (2016).

## Supplementary Material

Supplementary Information

## Figures and Tables

**Table 1:** Profiles of first sample set and second sample set.

	First sample set (resequencing stage)		Second sample set (genotyping stage)		
	SCZ	ASD	SCZ	ASD	CON
Total	574	152	4145	381	4432
Males (%)	56.6	80.3	52.8	77.4	45.2
Mean age ± SD^a^	52.4 ± 14.7	16 ± 7.9	47.9 ± 14.6	19.7 ± 10.8	43.6 ± 14

Abbreviations: SCZ, schizophrenia; ASD, autism spectrum disorders; CON, healty control; SD, standard deviation.

^a^Age at recruitement.

**Table 2:** Details of discovered rare missense mutations and *in-silico* analyses.

Chromosome^a^	Physical Position^a^	Exon	cDNA position^b^	Base Change M^c^ > m^c^	dbSNP Reference^d^	AA substitution	SCZ (n = 574)		ASD (n = 152)		In silico analyses for AA substitutions		
							Genotype count^e^	MAF	Genotype count^e^	MAF	Polyphen 2	SIFT	Pmut
12	13866157	2	c.52	G > A	rs201094029	V18I	0/0/279	N/A	0/2/150	0.0067	0.106 (benign)	0.14 (tolerated)	0.0439 (neutral)
12	13611737	8	c.1768	G > A	rs145021339	A590T	0/1/573	0.0009	0/0/150	N/A	0.987 (probably damaging)	0.158 (tolerated)	0.1209 (neutral)
12	13564120	13	c.3118	G > A	rs202222002	G1040S	0/0/571	N/A	0/1/149	0.0034	1.000 (probably damaging)	0.605 (tolerated)	0.3915 (neutral)
12	13563942	13	c.3296	G > A	N/A	R1099H	0/0/571	N/A	0/1/149	0.0034	0.999 (probably damaging)	0.016 (damaging)	0.4828 (neutral)
12	13563363	13	c.3875	A > G	N/A	K1292R	0/1/567	0.0009	0/0/150	N/A	0.014 (benign)	0.411 (tolerated)	0.0536 (neutral)

Abbreviations: SCZ, schizophrenia; ASD, autism spectrum disorders ; N/A, not applicable; MAF, minor allele frequency; AA, amino acid.

^a^Genomic position is based on GRCh38.

^b^cDNA position is based on ENST00000609686.

^c^M, major allele; m, minor allele.

^d^dbSNP release142.

^e^Genotype count; homozygote of major allele/heterozygote/homozygote of minor allele.

**Table 3:** Association analyses of missense variants.

Genomic data			SCZ (n = 4145)			ASD (n = 381)			CON (n = 4432)	
Variant	Position	M^a^/m^a^	MAF	Genotype count^b^	P value^c^	MAF	Genotype count^b^	P value^c^	MAF	Genotype count^b^
V18I	13866157	G/A	0.004	0/32/4078	1	0.0014	0/1/365	0.94	0.0041	0/35/4371
A590T	13611737	G/A	N/A	0/0/4106	0.5	N/A	0/0/363	1	0.0002	0/2/4403
G1040S	13564120	G/A	0.0003	0/2/4108	1	N/A	0/0/363	1	0.0004	0/3/4394
R1099H	13563942	G/A	0.0001	0/1/4117	0.96	N/A	0/0/363	1	0.0005	0/4/4399
K1292R	13563363	A/G	0.0004	0/3/4108	0.11	N/A	0/0/363	1	N/A	0/0/4393

Abbreviations: SCZ, schizophrenia; ASD, autism spectrum disorders ; N/A, not applicable; MAF, minor allele frequency.

^a^M, major allele; m, minor allele.

^b^Genotype count; homozygote of major allele/heterozygote/homozygote of minor allele.

^c^P values were calculated by Fisher’s exact test (2 × 2 contingency table, two-tailed).
