# Supplementary material for: Mutation screening of GRIN2B in schizophrenia and autism spectrum disorder in a Japanese population
Source: Sci Rep. 2016 Sep 12;6:33311. doi: 10.1038/srep33311 (PMC5018849; doi:10.1038/srep33311)
Supplement: Supplementary Information [file srep33311-s1.pdf]

Mutation screening of *GRIN2B* in schizophrenia and autism spectrum disorder in a Japanese population - Supplementary Information

Yuto Takasaki[1], Takayoshi Koide[1], Chenyao Wang[1], Hiroki Kimura[1], Jingrui Xing[1], Itaru Kushima[1,2], Kanako Ishizuka[1], Daisuke Mori[1,3], Mariko Sekiguchi[1,4], Masashi Ikeda[6], Miki Aizawa[6], Naoko Tsurumaru[6], Yoshimi Iwayama[7], Akira Yoshimi[1], Yuko Arioka[1,5], Mami Yoshida[1], Hiromi Noma[1], Tomoko Oya-Ito[1], Yukako Nakamura[1], Shohko Kunimoto[1], Branko Aleksic[8,*], Yota Uno[1], Takashi Okada[1], Hiroshi Ujike[9], Jun Egawa[10,11], Hitoshi Kuwabara[12], Toshiyuki Someya[10], Takeo Yoshikawa[7], Nakao Iwata[6], and Norio Ozaki[1].

[1]Department of Psychiatry, Nagoya University Graduate School of Medicine, Nagoya, Aichi, Japan

[2]Institute for Advanced Research, Nagoya University, Nagoya, Aichi, Japan

[3]Brain and Mind Research Center, Nagoya University, Nagoya, Aichi, Japan

[4]Department of Pharmacology, Nagoya University Graduate School of Medicine, Nagoya, Aichi, Japan

[5]Center for Advanced Medicine and Clinical Research, Nagoya University Hospital, Nagoya, Aichi, Japan

[6]Department of Psychiatry, Fujita Health University School of Medicine, Toyoake, Aichi, Japan

[7]Laboratory for Molecular Psychiatry, RIKEN Brain Science Institute, Wako, Saitama, Japan

[8]Department of Psychiatry, Nagoya University Graduate School of Medicine, Nagoya, Japan

[9]Department of Psychiatry, Ujike Nishiguchi Clinic (HU), Okayama, Japan

[10]Department of Psychiatry, Niigata University Graduate School of Medical and Dental Sciences, Niigata, Japan

[11]Department of Pediatric Psychiatry, Center for Transdisciplinary Research, Niigata University, Niigata, Japan

[12]Department of Child Neuropsychiatry, Graduate School of Medicine, The University of Tokyo, Tokyo, Japan

*Corresponding author: branko@med.nagoya-u.ac.jp

Table of contents

Supplementary results 1. Clinical Information for a K1292R Carrier

S Figure 1. Association between NR2B and CaMKII.

S Table 1. List of primer sets.

S Table 2. List of discovered synonymous mutations.

Supplementary results 1. Clinical Information for a K1292R Carrier

General data of patient:

A patient carrying the K1292R variant was a male diagnosed with SCZ at the age of 15. At the time of recruitment for this study, he was at the age of 41, 170 cm tall, weighed 74 kg. His mother gave birth to him when she was at the age of 27.

Childhood history:

The patient was a middle child, having an older brother and younger sister. He thrived without a significant episode of illness from birth until adolescence. He succeeded in passing a difficult entrance exam for a local high school at the age of 13.

History of illness:

He started to suffer from symptoms shortly after this, at the age of 15. Initial complaints were regarding general malaise and auditory hallucinations. He later began to talk to himself using disorganized speech. He was admitted to the hospital and was diagnosed with SCZ at the age of 18. After an in-patient admission lasting 1 month, he was discharged from the hospital. However, his medical compliance was poor, and he continued to talk to himself and yell at his family members. He used violence against his family. He sent to a psychiatric hospital again due to his state of severe hallucinations and bizarre delusions. After hospitalization, severe psychosis was relieved, but his minor delusions and social withdrawal were still present.

Family history:

His father had not diagnosed for any psychiatric condition, however, he committed suicide when he was 14 years old. Although we do not have detailed clinical information about patient's father, is should be noted, there is a significant body of evidence indicating that >80% of suicides

occur in people with a clinically diagnosable psychiatric disorder[1, 2]. Subsequently, the father also may have had some form of mental illness, although we cannot unequivocally confirm that. Other members of the family did not have a significant past medical history.

Medications:

He was taking antipsychotic drugs equivalent to 1400 mg chlorpromazine at the time of requirement for this study.

S Figure 1. Association between NR2B and CaMKII.

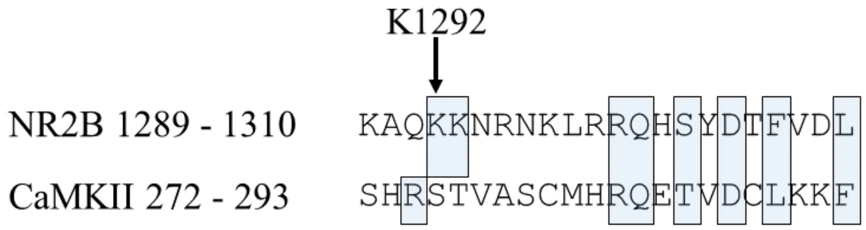

T-site interaction residues of CaMKII are shown in blue. In the inactive state of CaMKII, this site is covered by self-alignment around T286, thereby inhibiting the kinase activity. In the active state, Ca^{2+}/CaM displaces this auto-inhibition. The T-site is subsequently occupied by a residue of NR2B. This association allows successive kinase activity of CaMKII[3].

S Table 1. List of primer sets.

Exon	Forward primers	Reverse primers	Primers for cycle sequence reaction
2	GTGCTCAATGAAAGGAGATAAGGTC	GTAGAGAAATCACCTGCCTTGAG	
3	GGACCACTGAGCTTATCTCACC	TCAACCTGCTACCGTCTTGAA	
4	TGAGAACATGCGGTGACCAA	ATGGCTTCTCCTGTGTATCAAGGT	
5	CATGGGTCTTAACAGGGTGC	CACATGCTGACTAAATGGCAGGA	CATGGGTCTTAACAGGGTGC
6	CATGGGTCTTAACAGGGTGC	CACATGCTGACTAAATGGCAGGA	AAATTGTGCTGAGCTGTGAAG
7	CATGGGTCTTAACAGGGTGC	CACATGCTGACTAAATGGCAGGA	CACATGCTGACTAAATGGCAGGA
8	CCTTGACCTTTAAGGAAATGGTTCAGTACA	ATTTCTAAAGAGACGCCAAGCTGGTGA	
9	CCCTGTGTATTTGTAGGTTTCAACAGA	GGAAACTGACTTCTACTCCCATGTTC	
10	ACATTCCTTTGGCCCATCAA	GGAAGAGATTGACCCTGCTG	
11	ACATTCCTTTGGCCCATCAA	GGAAGAGATTGACCCTGCTG	CATATTAATGGCCCCACAGG
12	TCATATCGCCTGCGGTACTC	TACAGAGAACCAACTGCCCA	
13	ACCTCAGCTCACCACATGACAGTC	CAGTAGGAACCAGAACTCCAGGATC	GCAACGTGTACCAAGATCACTACC,<br>TGGACCAGTTCCGAACAAAGG,<br>AGTACCCTCAGAGCCCGACTA

Note: Exon 1 has not been sequenced, because it only encodes the 3′UTR. Exons 5-6 and Exons 10-11 were amplified at once. After amplification, they were sequenced with discrete primers in the cycle sequence reaction. Exon 13 was sequenced with four primers because it is over 2 kb in length.

S Table 2. List of discovered synonymous mutations.

Chromosome[a]	Physical position[a]	Exon[b] /Intron[b]	cDNA position[b]	Base Change M^c>m^c	Observed number[d]	
					SCZ (n = 580)	ASD (n = 168)
12	13753709	Exon 3	c.609	C>T	3	1
12	13753556	Exon 3	c.771	C>T	3	1
12	13615130	Exon 7	c.1638	A>C	2	N/A
12	13611840	Exon 8	c.1665	C>T	1	N/A
12	13611716	Intron 8	c.1780+9	G>A	6	1
12	13571793	Intron 10	c.2171+11	C>T	2	1
12	13567133	Exon 12	c.2490	T>C	1	N/A
12	13411709	Intron 12	c.2659-4	T>C	5	N/A
12	13564607	Exon 13	c.2631	C>T	1	N/A
12	13564469	Exon 13	c.2769	C>T	1	N/A
12	13564007	Exon 13	c.3231	C>T	1	N/A
12	13563578	Exon 13	c.3660	C>G	3	1
12	13563323	Exon 13	c.3915	C>T	1	N/A
12	13563002	Exon 13	c.4236	G>A	N/A	1

Abbreviations: SCZ, schizophrenia; ASD, autism spectrum disorders; N/A, not applicable.

[a] Genomic position is based on GRCh38.

[b] cDNA position is based on ENST00000609686.

[c] M, major allele; m, minor allele.

[d] Genotype count; count indicates the number of heterozygous samples.

Reference

1 Tidemalm, D. *et al.* Familial clustering of suicide risk: a total population study of 11.4 million individuals. *Psychological medicine* **41**, 2527-2534, doi:10.1017/S0033291711000833 (2011).

2 Nock, M. K., Hwang, I., Sampson, N. A. & Kessler, R. C. Mental disorders, comorbidity and suicidal behavior: results from the National Comorbidity Survey Replication. *Mol Psychiatry* **15**, 868-876, doi:10.1038/mp.2009.29 (2010).

3 Bayer, K. U., De Koninck, P., Leonard, A. S., Hell, J. W. & Schulman, H. Interaction with the NMDA receptor locks CaMKII in an active conformation. *Nature* **411**, 801-805, doi:10.1038/35081080 (2001).
